# Effects of safinamide on pain in patients with fluctuating Parkinson's disease

**DOI:** 10.1002/brb3.2336

**Published:** 2021-09-03

**Authors:** Sotirios Grigoriou, Pablo Martínez‐Martín, K. Ray Chaudhuri, Katarina Rukavina, Valentina Leta, Denise Hausbrand, Björn Falkenburger, Per Odin, Heinz Reichmann

**Affiliations:** ^1^ Division of Neurology Department of Clinical Sciences Lund Lund University Lund Sweden; ^2^ Department of Neurology and Rehabilitation Medicine Neurology Skåne University Hospital Sweden; ^3^ National Center of Epidemiology and CIBERNED Carlos III Institute of Health Madrid Spain; ^4^ Department Basic and Clinical Neuroscience The Maurice Wohl Clinical Neuroscience Institute King's College London Cutcombe Road London UK; ^5^ Parkinson's Foundation Centre of Excellence King's College Hospital Denmark Hill London UK; ^6^ Department of Neurology University Hospital Carl Gustav Carus Technische Universität Dresden Dresden Germany

**Keywords:** glutamate, MAO‐B inhibitor, non‐motor symptoms, pain, Parkinson's disease, safinamide

## Abstract

**Background:**

Non‐motor symptoms (NMS) are integral to Parkinson's Disease (PD) and management remains a challenge. Safinamide is a novel molecule in relation to addressing NMS due to its multifocal mechanism of action with both dopaminergic and non‐dopaminergic properties.

**Objective:**

To investigate the efficacy of safinamide on NMS and its burden in PD patients with motor fluctuations after 6 months of treatment.

**Methods:**

This observational, multicenter, open‐label, pilot study assessed a wide range of NMS using the following rating scales, NMSS (non‐motor symptom scale), KPPS (King's PD pain scale), HADS (hospital anxiety and depression scale), PDQ‐8 (Parkinson's disease quality of life questionnaire), and PDSS‐2 (Parkinson's disease sleep scale), EuroQol‐5D 3 level version (EQ‐5D‐3L), CGI‐I (clinical global impression of improvement), and PGI‐C (patient global impression of change). Motor examination using UPDRS part III (Unified Parkinson's disease rating scale, motor examination), UPDRS IV (complications of therapy) and Hoehn and Yahr staging were also obtained.

**Results:**

27 patients were included in the analysis and were evaluated at baseline and ≥ 6 months after safinamide treatment. 26 patients had a daily maintenance dose of 100 mg and 1 patient a daily dose of 50 mg. Significant improvements in UPDRS IV, KPPS item 5 (region‐specific “off” dystonia), KPPS domain 3 (items 4–6, fluctuation related pain) and KPPS total score were observed after treatment with safinamide, while maintaining stable dopaminergic medication. No statistically significant differences were found in NMSS, HADS, PDSS‐2, EQ‐5D‐3L, and PDQ‐8 after treatment.

**Conclusions:**

Our results suggest that safinamide may have a beneficial effect on pain, a key unmet need in fluctuating PD patients.

## Graphical abstract

In this observational prospective study we investigate the effects of safinamide on non‐motor symptoms in patients with fluctuating Parkinson's disease. Our main finding is a significant improvement in UPDRS IV and pain as measured by the KPPS.

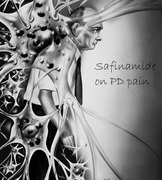

## 1 INTRODUCTION

The classic findings of Parkinson's disease (PD) are motor and resultant from dopamine deficiency within the basal ganglia (Kalia and Lang, 2015), while non‐motor symptoms (NMS) have a more complex pathophysiology and can range from the prodromal to the palliative stage. It is common for patients with advanced PD to experience a number of NMS (neuropsychiatric problems, sleep disorders, pain, autonomic symptoms including gastrointestinal and urogenital etc.), while some NMS already appear in the preclinical phase of the disease, such as, anosmia and rapid eye movement sleep behavior disorder (RBD) (Chaudhuri and Odin, 2010; Pont‐Sunyer et al., 2015; Poewe, 2008; Gallagher et al., 2010; Maass and Reichmann, 2013). The pathophysiology of NMS is multifactorial and it is believed that dysfunction of both dopaminergic and non‐dopaminergic systems contribute to their development. Outside of the nigrostriatal pathway, α‐synuclein can have a diverse neuroanatomical distribution that differs from patient to patient, which likely accounts for different non‐motor manifestations in PD patients (Chaudhuri and Schapira, 2009; Lang and Obeso, 2004; Barone, 2010; Schapira et al., 2017; Adler and Beach, 2016). NMS have a significant cumulative effect on patients’ daily activities and quality of life (Kadastik‐Eerme et al., 2016). Studies have shown that NMS, as a whole, may have a greater impact on the patients’ quality of life than motor symptoms and that the NMS progression contributes to further decline of the quality of life in PD patients (Gallagher et al., 2010; Martinez‐Martin et al., 2011).

MAO‐B inhibitors prevent the breakdown of dopamine and stabilize dopamine concentrations in the synaptic cleft, prolonging the effects of dopamine (Szökő et al., 2018). Due to their mechanism of action they offer a limited amelioration of impaired motor behavior and wearing‐off phenomena in PD patients (Riederer and Müller, 2018). They are indicated for initiation of treatment in patients with mild PD symptoms before the initiation of levodopa, or mainly as an add‐on treatment in PD patients with fluctuations at a later stage (Overview, 2017). MAO‐B inhibitors currently available for PD treatment are selegiline, rasagiline, and safinamide.

Safinamide (Xadago) is an orally active, selective, reversible MAO‐B inhibitor and is approved for the treatment of mid‐ to late‐stage fluctuating PD as an add‐on to other PD medications. Safinamide is administered daily as a single dose of 50 or 100 mg (Blair and Dhillon, 2017). It has a unique mechanism of action that includes both dopaminergic and non‐dopaminergic properties, as it leads to an inhibition of glutamate release by modulation of calcium‐ and sodium ion channels (Caccia et al., 2006; Müller and Foley, 2017). Numerous studies have shown that safinamide has a positive effect on motor symptom control, ON‐time and fluctuations in both early and advanced PD, having a dopamine‐sparing effect, as well as, being generally well tolerated (Cattaneo et al., 2016; Borgohain et al., 2014; Schapira et al., 2017; Stocchi et al., 2012; Barone et al., 2013; Borgohain et al., 2014; Hattori et al., 2020). Safinamide's compound dopaminergic and non‐dopaminergic properties make it an interesting agent in the field of NMS and neurodegeneration in PD (Pisanò et al., 2020; Maiti et al., 2017; Zhang et al., 2016; Sadeghian et al., 2016) and especially pain, that has previously been associated with high glutamatergic activity (Watson, 2016; Bleakman et al., 2006; Phillips and Clauw, 2011).

The objective of this exploratory pilot study was to investigate how the addition of safinamide in fluctuating PD patients, while keeping an otherwise stable antiparkinsonian treatment, affects different NMS. This was performed in a multicenter, open‐label, observational study in an outpatient clinic setting.

## 2 MATERIALS AND METHODS

### 2.1 Study design

The study is a collaboration between Skane University Hospital in Lund, Sweden, Dresden University Hospital, Germany, Parkinson Foundation International Centre of Excellence, King's College Hospital, London, United Kingdom and National Center of Epidemiology and CIBERNED, Carlos III Institute of Health, Madrid, Spain. Patient recruitment took place in Dresden and Lund.

It is an investigator‐initiated, observational pilot study on safinamide's effect on NMS in fluctuating PD patients. Patients with idiopathic PD were evaluated before (Visit 1, V1) and at least 6 months following the initiation of safinamide (Visit 2, V2) with the patients being mainly included from outpatient clinics. The patient's clinician was responsible for initiating and eventually continuing treatment with safinamide according to common clinical practice and with indication of motor symptom improvement/ off time reduction, while trying to maintain a stable treatment with levodopa and other antiparkinsonian agents (dopamine agonists, COMT‐inhibitors, amantadine, anticholinergics). Patients that were already on selegiline or rasagiline before the study went through a wash‐out period of 4 weeks before the study start as per routine clinical practice, discontinuing their MAO‐B inhibitor treatment. Patients on MAO‐A inhibitors were not included in this study.

### 2.2 Patients

Patients were included according to the following inclusion criteria: an age of 30 to 90 years; a diagnosis of idiopathic PD; a Hoehn and Yahr stage of I‐IV during “off” phase; motor fluctuations, with >1.5 h “off” time during the day; and have been receiving treatment with a stable dose of levodopa for at least 4 weeks. The exclusion criteria were the same as in the safinamide SETTLE study (Schapira et al., 2017). Safinamide was initiated at a 50 mg dose once daily and was up‐titrated to a maintenance dose of 100 mg once daily for 26 of the 27 patients, while one patient chose to remain on a maintenance dose of 50 mg once daily throughout the study.

**FIGURE 1:**
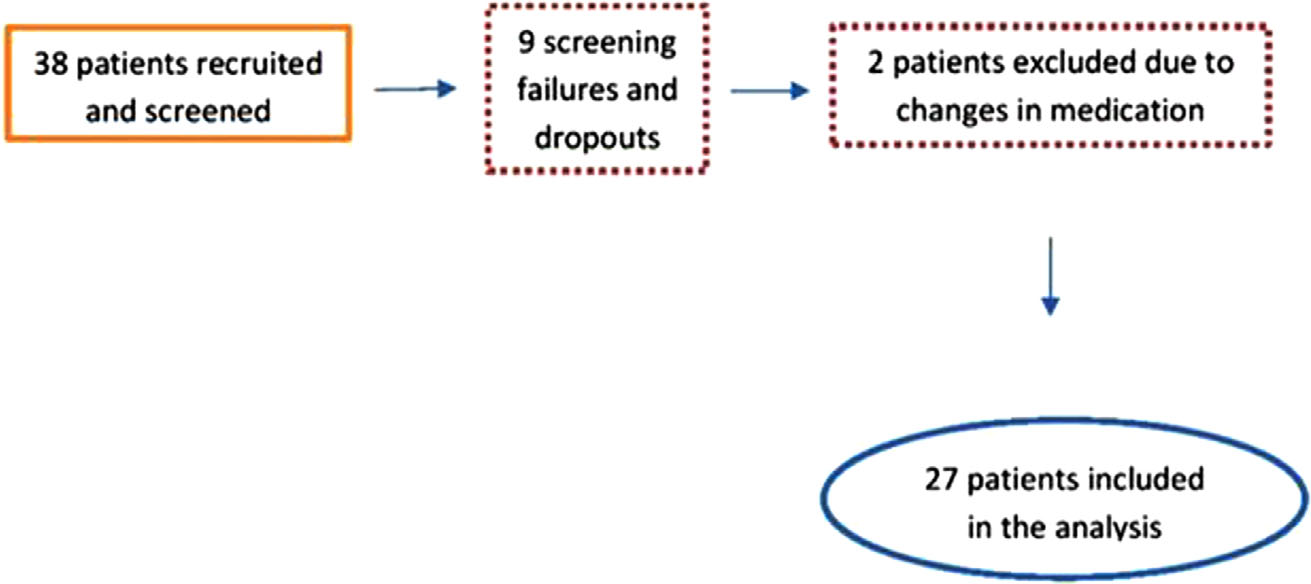
Patient flow chart

We recruited 38 patients; 5 patients were screening failures and 4 dropouts (early discontinuation due to loss of interest in completing the study/ no significant subjective motor improvement in comparison to rasagiline/ medication cost), while 2 patients were excluded from the statistical analysis because of changes in their anti‐parkinsonian medications during the study. The remaining 27 patients were included in the final analysis (Figure 1).

### 2.3 Assessments

The patients were evaluated using well‐known, validated and broadly utilized scales to assess the effects of treatment with safinamide in a broad spectrum of NMS. Sociodemographic data, other antiparkinsonian treatment, including calculation of Levodopa equivalent daily doses (LEDD), and PD‐related history were also collected. The LEDDs were calculated according to the literature (Tomlinson et al., 2010; Schade et al., 2020).

The scales and questionnaires used to assess the NMS and quality of life were: NMSS (non‐motor symptom scale) (Chaudhuri et al., 2007), HADS (hospital anxiety and depression scale) (Snaith, 2003), PDQ‐8 (Parkinson's disease quality of life questionnaire) (Jenkinson et al., 1997), and PDSS‐2 (Parkinson's disease sleep scale) (Trenkwalder et al., 2011), EuroQol‐5D 3 level version (EQ‐5D‐3L) (Brooks, 1996), CGI‐I (clinical global impression of improvement), and PGI‐C (patient global impression of change) (Guy, 1976). UPDRS part III (unified Parkinson's disease rating scale, motor examination), UPDRS IV (complications of therapy) (Fahn, 1987) and Hoehn and Yahr staging were also obtained. Motor examination UPDRS part III and Hoehn and Yahr staging were performed during “on” phase, as recommended by the MDS taskforce.

Pain was assessed prior to and while on safinamide treatment using the King's PD pain scale (KPPS), a 14‐item, PD‐specific pain scale. Each item is scored by severity (0–3) multiplied by frequency (0–4), resulting in a sub‐score of 0 to 12. There is a total possible score range from 0 to 168, and pain is classified into 7 different domains (Domain 1: Item 1, Domain 2: Item 2–3, Domain 3: Item 4–6, Domain 4: Item 7–8, Domain 5: 9–11, Domain 6: Item 12–13, Domain 7: Item 14) (Chaudhuri et al., 2015). The English version of KPPS was used for patient evaluation in both Germany and Sweden.

### 2.4 Statistical analysis

Statistical analyses were performed using IBM SPSS Statistics for Windows, Version 26.0. Armonk, NY: IBM Corp. Shapiro‐Wilk test was performed to assess the normality of value distribution for the different variables/ items. For data that were not normally distributed, the related‐samples Wilcoxon signed rank test was used to try and detect statistically significant differences in the separate items of each scale, as well as in sub‐total and total scores. Paired samples t‐test was used for data with normal distribution. The significance level used was *p* < .05. The correlations between the baseline and follow‐up differences in KPPS scores and UPDRS item 39 were calculated using the Spearman's rank order correlation coefficient. The concordance between the global impression of change from baseline to follow‐up by patients and clinicians (PGI‐C and CGI‐C) was tested by means of percentage of agreement and weighted kappa with quadratic weights.

### 2.5 Ethics

The study was approved by the Swedish Ethical Review Authority and the Ethical Committee of Dresden, Germany. All patients signed an informed consent form and the study was conducted according to good clinical practice rules and to the Declaration of Helsinki.

## 3 RESULTS

Patients’ demographic and clinical characteristics are summarized in Table 1.

**TABLE 1:** 

**Patient characteristics**		Range
**Age** (years)	Mean: 65	38–87
**Gender**	Male: 22	
	Female: 5	
**PD duration** (years)	Mean: 6.8	1–20
**Hoehn & Yahr**	Median: 2.5	1–4
**Follow‐up** (months)	Mean: 6.6	6–9
**LEDD** mean (mg)	Baseline: 963	400–1890
	Follow‐up: 955	

The majority of the patients were male (22; 81%) and the mean age of the patients was 65 years. The PD duration was calculated with PD diagnosis as a start point, although most of the patients reported PD‐related symptoms (motor and non‐motor) months to years before the diagnosis was set. The mean PD duration from diagnosis to inclusion was 6.8 years. All Hoehn & Yahr (H&Y) stages, except stage 5, were represented in the study population, with a median H&Y stage of 2.5. The majority of patients (22 of 27) had H&Y stage of 2 and 3. The mean follow‐up time was 6.6 months with a range of 6–9 months after treatment initiation. The first patient was included in July 2018 and the last patient completed the study in January 2020.

All patients remained on the same antiparkinsonian agents during the study. 19 patients had no changes in their levodopa equivalent daily dose (LEDD), 5 patients had <10% LEDD change and 3 patients 10–20% change at the end of the study. The mean LEDD at baseline was 963 mg (SD ± 76 mg) and 955 mg (SD ± 75 mg) at follow‐up, showing a high level of compliance with retaining a stable medication during the study. The calculation of LEDD at follow‐up was performed for all other antiparkinsonian medication not taking safinamide into account.

**FIGURE 2:**
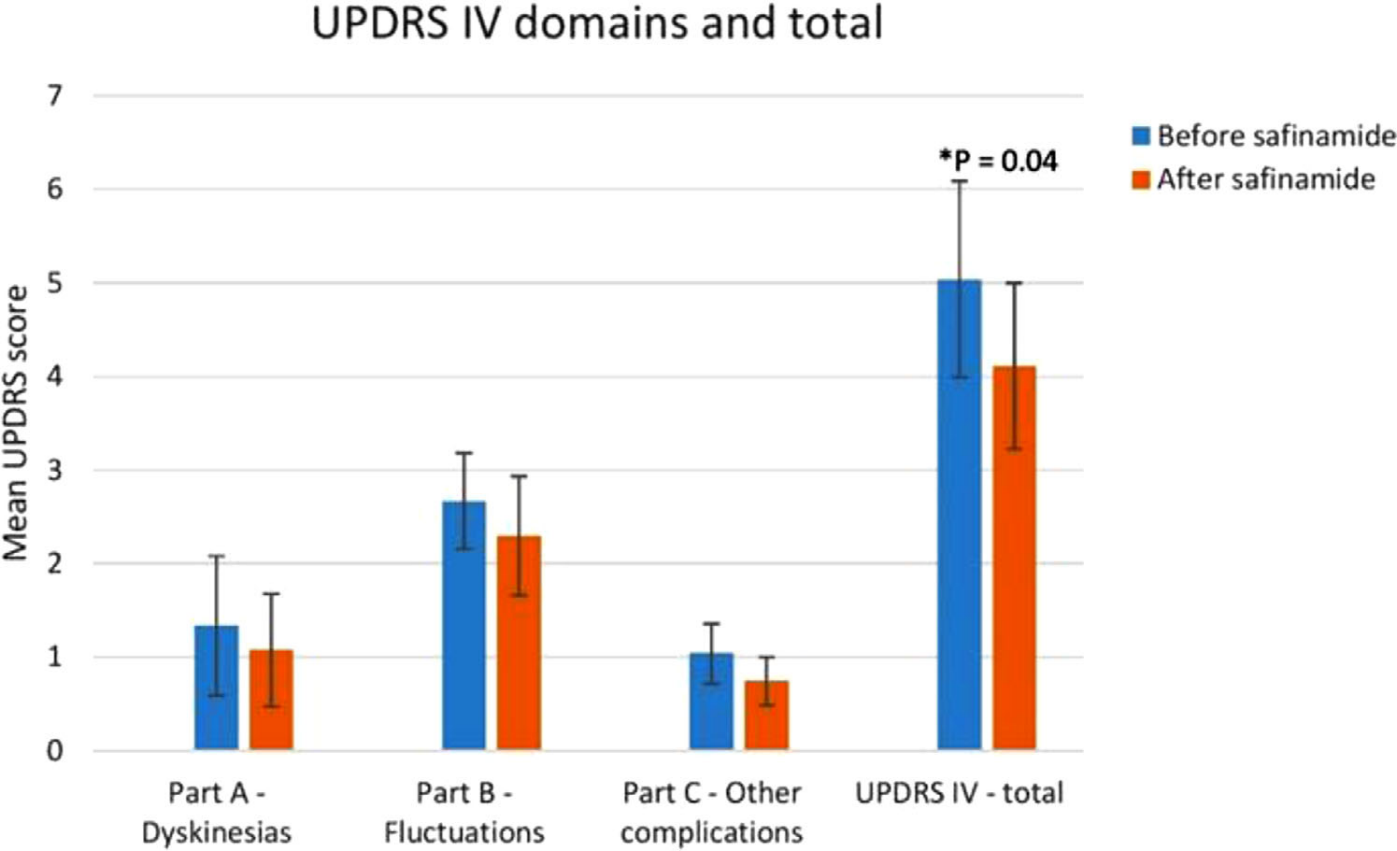
Changes in UPDRS IV domains and total score, evaluated before and after ≥ 6 months of treatment with safinamide. Part A: UPDRS items 32–35, Part B: UPDRS items 36–39, Part C: UPDRS items 40–42. Error bars represent 95% confidence interval

Although safinamide's effect on NMS was the main focus of the study, an evaluation of the UPDRS III and IV was performed before and after treatment. The mean UPDRS III score was 21.9 (range: 10–52) at baseline and 21.7 (range: 4–48) at follow‐up, showing no statistically significant difference. A statistically significant improvement, *p* = .04, was found in the total UPDRS IV score. The mean UPDRS IV at baseline was 5 (range: 1–13) and at follow‐up 4.1 (range: 0–9), as shown in Figure 2.

A non‐significant (*p* = .29) reduction was observed in the mean NMSS total score, from 60.6 at baseline to 57.1 at follow‐up. None of the NMSS domains or separate items showed any statistically significant improvement, Table 2.

**TABLE 2:** 

	**Baseline (mean ± SD)**	**Follow‐up (mean ± SD)**	**P value**
NMSS	60.6 (±31.2)	57.1 (±34.6)	0,29 (NS)
PDSS‐2	14.8 (±7.4)	13.8 (±8.2)	0.35 (NS)
HADS anxiety	5.2 (±3.7)	4.8 (±2.9)	0.50 (NS)
HADS depression	5.0 (±4.0)	4.7 (±3.5)	0.70 (NS)
PDQ‐8	30.1 (±18.1)	30.1 (±18.3)	0.89 (NS)
EQ‐5D‐3L	0.67 (±0.23)	0.72 (±0.19)	0.22 (NS)

*Note*: NMSS and PDSS‐2 represent total scores; PDQ‐8 refers to PDQ‐8 summary index; EQ‐5D‐3L represents time trade‐off (TTO).

Abbreviations: NS, non‐significant.

**FIGURE 3:**
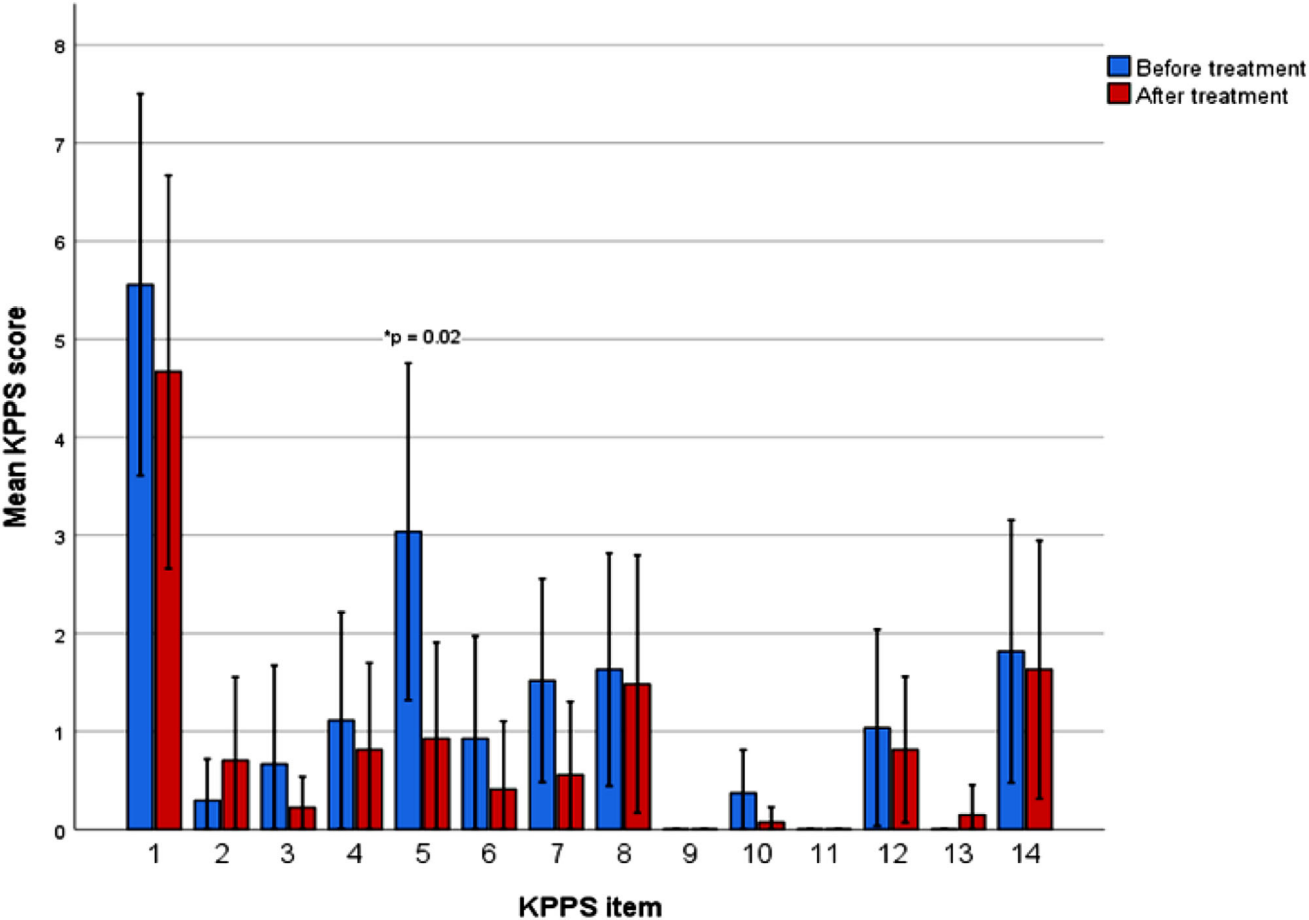
Changes in KPPS items, evaluated before and after ≥ 6 months of treatment with safinamide. X axis represents the different KPPS items/questions, clustered by time before and after treatment and Y axis the mean score of each KPPS item. Error bars represent 95% confidence interval

**FIGURE 4:**
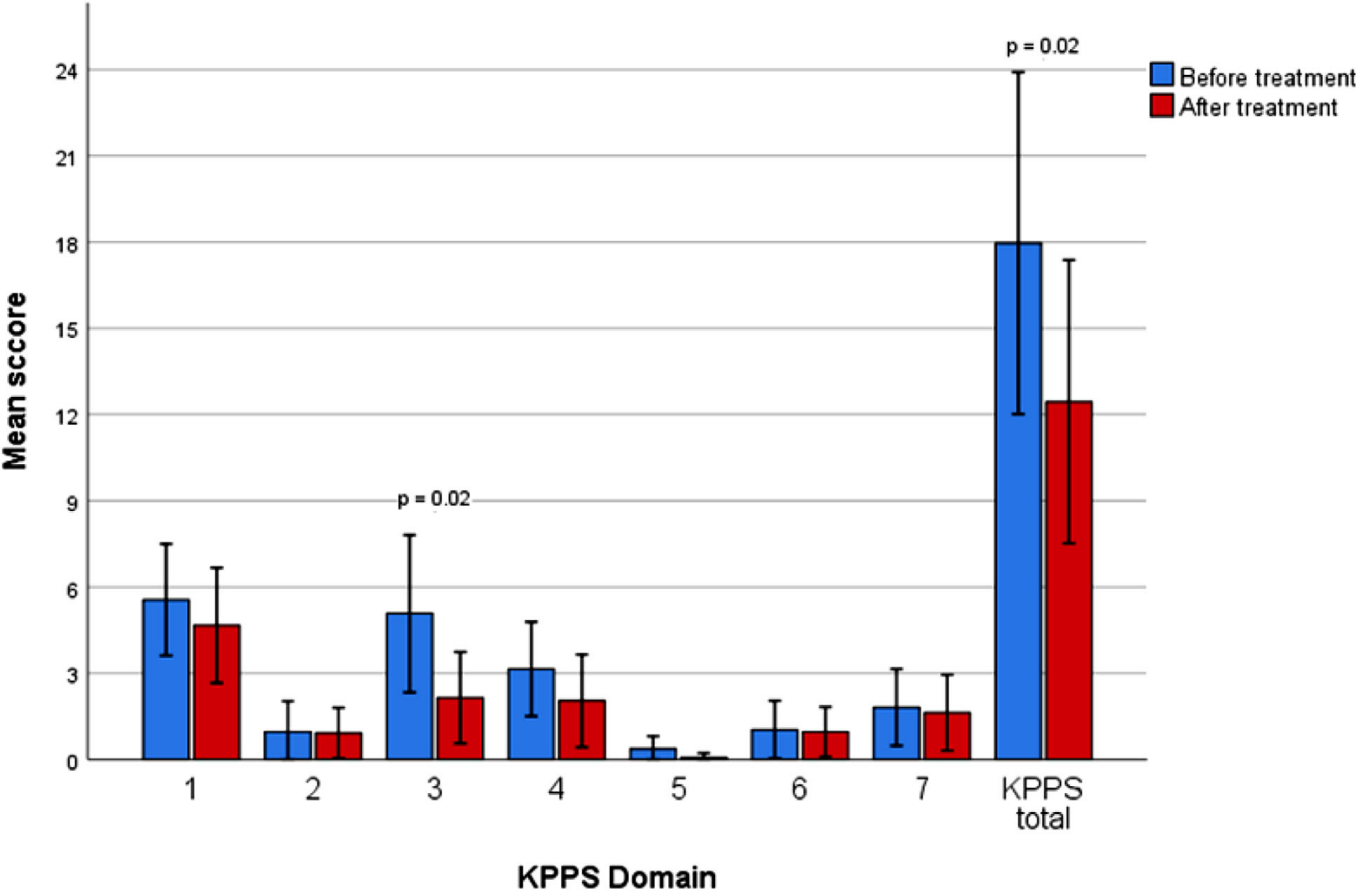
Changes in KPPS domains and total, before and after ≥ 6 months of treatment with safinamide. X axis represents the different KPPS items/questions, clustered by time before and after treatment and Y axis the mean score of each KPPS item. Error bars represent 95% confidence interval. Domain 1: Musculoskeletal pain, 2: Chronic pain, 3: Fluctuation‐related pain, 4: Nocturnal pain, 5: Orofacial pain, 6: Discolouration; Oedema/Swelling, 7: Radicular pain

The analysis of each KPPS item separately revealed a significant improvement in KPPS item 5 (“Off” dystonia in a specific region), which was reduced from 3.0 at baseline to 0.9 at follow up, *p* = .02. KPPS Domain 3 (items 4–6, fluctuation‐related pain) improved significantly from a mean of 5.1 to 2.1 (*p* = .02). The mean total KPPS score showed a statistically significant improvement, *p* = .02, from 18.0 at baseline to 12.4 at follow‐up (‐5.6 points, 31.1 % improvement). The KPPS statistics are presented in Figures 3 and 4.

The differences noted in the KPPS item 5 and KPPS domain 3 between baseline and follow‐up had a low correlation with the difference in UPDRS item 39 (percentage time in off) with a Spearman's correlation coefficient of 0.23 and 0.21, respectively. The difference in KPPS total score had also a low level of correlation with the difference in UPDRS item 39, with a correlation coefficient of 0.16. The p values of these correlations though did not reach statistical significance (*p* > .05).

A non‐significant change was seen in PDSS‐2 which was used to assess sleep problems (mean total PDSS‐2 score of 14.8 at baseline and 13.8 at follow‐up, *p* = .35). No significant changes were found between baseline and follow‐up for HADS‐depression (5.0 – 4.7), HADS‐anxiety (5.2 – 4.8), PDQ‐8 summary index (30.1 – 30.1), and EQ‐5D‐3L time trade‐off index value (TTO; 0.67 – 0.72), Table 2.

PGI‐C and CGI‐C scales showed a very strong agreement between the patients’ and the clinicians’ global impression with a high kappa coefficient value, Kappa (95% CI) = 0.87 (0.73 – 0.93). 10 patients improved at follow‐up according to both PGI and CGI, while 5 patients had worse scores in both scales at follow‐up. No change was noticed in 7 patients (both scales) and the rest of the cases showed a discrepancy between clinician‐patient rating (PGI‐CGI).

## 4 DISCUSSION

Several studies have looked at safinamide's effect on NMS. A recent retrospective observational study with 20 participants on stable antiparkinsonian treatment has shown a significant reduction in the cardiovascular, sleep/fatigue, mood/cognition, attention/memory, urinary, and sexual function domains of the NMSS, after at least three months of safinamide treatment. No significant differences were observed in the Mini Mental State Examination (MMSE), Hospital Anxiety and Depression Scale (HADS) or PD Sleep Scale (PDSS) (Bianchi et al., 2019). Another observational study focusing on sleep disturbances of fluctuating PD patients has, on the other hand, shown significantly reduced scores in PDSS‐2 (by approx. 3 points) and Epworth Sleepiness Scale (ESS) after four months of safinamide treatment (Liguori et al., 2018), while in our study, we could only detect a non‐significant reduction in the total PDSS‐2 score. This finding could be attributed to the fact that the previously mentioned study included patients with a higher baseline PDSS‐2 score than in our study (20.1 vs. 14.8), the difference in patient sample size (47 vs. 27) and the different study designs.

Post‐hoc analyses from previous studies have shown positive effects of safinamide on mood and emotional well‐being (Cattaneo et al., 2017), as well as improvements in PD quality of life questionnaire (PDQ‐39) questions related to pain, and a reduction of pain medications (Cattaneo et al., 2017; Cattaneo et al., 2018). A recent, prospective, observational study with 13 patients that focused on safinamide and pain also indicates a positive effect after 12 weeks of treatment (Geroin et al., 2020).

The results of our study also suggest a positive effect of safinamide in pain in PD patients, as has been reported in the studies mentioned above (Cattaneo et al., 2017; Geroin et al., 2020). In the 2 post‐hoc analyses (Cattaneo et al., 2017; Cattaneo et al., 2018) at 6 months and 2 years after safinamide treatment respectively, the investigators observed a 24% and 26% reduction of pain medications, which aligns well with the 31% reduction in KPPS total score we found at 6 months in our cohort. The recent Italian study on safinamide and pain (Geroin et al., 2020) found an even more profound reduction in KPPS total score (approx. 50%), but had a shorter follow‐up at 12 weeks and included patients with much higher pain burden (more than double KPPS total score at baseline) in comparison to our patient cohort.

In our patient group we were also able to show a significant improvement in the total KPPS score, as well as in the item 5, related to region‐specific “off” dystonia. KPPS Domain 3 (items 4–6, fluctuation related pain) was also significantly improved, with items 4 and 6 showing an improvement, though not statistically significant as separate items. Our results indicate a low correlation of the improvement observed in KPPS item 5, KPPS domain 3, and KPPS total score and the changes in UPDRS item 39. This could suggest that pain improvement may not exclusively arise due to secondary reduction of motor fluctuation but can perhaps be attributed to safinamide's double (dopaminergic and anti‐glutamatergic) mechanism of action. It should be kept in mind though that, probably due to the relatively small sample size, the Spearman's correlations did not reach statistical significance at the 0.05 level in our analysis. In order to prove this statement, further studies with a design that includes control groups (e.g. a PD group initiating safinamide with a comparable dose increment of dopaminergic medication or a control group treated with selegiline or rasagiline) are needed.

UPDRS part IV total score was also significantly reduced, with improvement (not statistically significant separately) noticed in all three parts of UPDRS IV (mainly Part A—fluctuations and Part C—other complications). While the UPDRS part III did not change significantly from baseline to follow up, this could be regarded in a positive light as the PD motor symptoms tend to deteriorate linearly as PD progresses (Holden et al., 2018). It should also be noted that while the UPDRS III assessments were performed in “on,” they were not obtained at the same timepoints at baseline and follow‐up.

We were not able to detect any other significant positive effects on NMS with the other scales used in the study. Total NMSS score showed only a trend of improvement, which however did not reach statistical significance and no changes could be detected in the patients’ quality of life or general health status (as measured by PDQ‐39 and EQ‐5D‐3L).

The main limitations of this study are the lack of blinding and randomization as it is an open‐label, observational study, although the data was gathered prospectively in a multicenter setup. Pain is a symptom known for its susceptibility to placebo effect and effects on it should thus be interpretated cautiously in studies without a placebo control group. Another limitation is the relatively small sample size as safinamide is not the first choice among MAO‐B inhibitors in Sweden, the UK and Germany. The study group consisted of consecutively recruited patients that were mainly male (22 of 27). The English version of KPPS was used in Sweden and Germany (the two centers that contributed with patient inclusion), as no linguistic validated translations of the scale were available at the time. This could lead to translation differences on different study visits, although the scale was applied by the same rater each time. Furthermore, pain symptoms and the way they are described are subjective and may be subject to discrepancies between different countries and cultures.

The main strengths of the study are the utilization of validated, well‐known clinical scales and measurements, its prospective nature and a patient group that was able in an observational setting to retain a stable antiparkinsonian treatment during the study. Furthermore, all evaluations were performed by the same clinician in each participating center at both visits, thus avoiding the issue of inter‐rater variability.

## 5 CONCLUSION

Safinamide is an interesting agent for the treatment of PD and NMS due to its dopaminergic and non‐dopaminergic mechanism of action. Our study suggests that it seems to have a beneficial effect on pain, mainly fluctuation‐related pain. Randomized trials with bigger patient populations as well as post marketing surveillance based studies are needed to confirm the effect of safinamide on different NMS, for example, pain, or detect other possible effects.

## AUTHOR CONTRIBUTIONS

Grigoriou Sotirios: Research project conception, data collection, statistical analysis, writing of the first draft of the manuscript. Martínez‐Martín Pablo, Chaudhuri K. Ray, Hausbrand Denise, Falkenburger Björn, Odin Per, Reichmann Heinz: Research project conception, data collection, review of the manuscript. Rukavina Katarina and Leta Valentina: Review of the manuscript.

## COMPETING INTEREST STATEMENT

The study was funded by Zambon SpA with an unconditional research grant. In addition, the study was funded by MultiPark, the strategic research area for neuroscience at Lund University; the Swedish Parkinson Foundation; the Swedish Parkinson Academy and the Faculty of Medicine at Lund University.

Grigoriou Sotirios has received academic grants from: Multipark, Elsa Schmitz foundation and O Stoltz foundation.

Martínez‐Martín Pablo has received honoraria from Editorial Viguera for lecturing in courses or publications; from Britannia for writing of an article; from the International Parkinson and Movement Disorder Society (IPMDS) for management of the Program on Rating Scales; and from Bial for advice in a clinical‐epidemiological study. Grants have been received from the IPMDS for development and validation of the MDS‐NMS.

Chaudhuri K. Ray has acted on advisory board for AbbVie, UCB, GKC, Bial, Cynapsus, Novartis, Lobsor, Stada, Medtronic, Zambon, Profile, Sunovion, Roche, Therevance, Scion and Britannia, and has received honoraria for lectures from AbbVie,Britannia, UCB, Mundipharma, Zambon, Novartis, Boeringer Ingelheim, and grants (Investigator Initiated) from Britania Pharmaceuticals, AbbVie, UCB, GKC, Bial, Academic grants: EU, IMI EU, Horizon 2020, Parkinson's UK, NIHR, PDNMG, EU (Horizon 2020), Kirby Laing Foundation, NPF, MRC, Welcome Trust.

Rukavina Katarina is supported by NIHR BRC.

Leta Valentina has received grants from BRC, Parkinson's UK, a travel and congress grant from Bial UK Ltd, speaker‐related activities fees from Britannia pharmaceuticals, and consultancy fees from Invisio Pharmaceuticals, outside the submitted work.

Odin Per has acted on advisory board for AbbVie, GKC, Bial, Lobsor, Zambon, and Britannia, and has received honoraria for lectures from AbbVie, Britannia, UCB, Zambon, and grants (Investigator Initiated) from AbbVie. Academic grants: Swedish Parkinson Foundation, Lund Medical Faculty, Region Skåne, Åhlens foundation.

Reichmann Heinz has received compensation from Zambon for lectures and advisory board meetings.

### PEER REVIEW

The peer review history for this article is available at https://publons.com/publon/10.1002/brb3.2336

## Data Availability

The data that support the findings of this study are available on request from the corresponding author. The data are not publicly available due to privacy or ethical restrictions.
